# A microdeletion del(12)(p11.21p11.23) with a cryptic unbalanced translocation t(7;12)(q21.13;q23.1) implicates new candidate loci for intellectual disability and Kallmann syndrome

**DOI:** 10.21203/rs.3.rs-2572736/v1

**Published:** 2023-03-27

**Authors:** Afif Ben-Mahmoud, Shotaro Kishikawa, Vijay Gupta, Natalia T. Leach, Yiping Shen, Oana Moldovan, Himanshu Goel, Bruce Hopper, Kara Ranguin, Nicolas Gruchy, Saskia M Maas, Yves Lacassie, Soo-Hyun Kim, Woo-Yang Kim, Bradley J. Quade, Cynthia C. Morton, Cheol-Hee Kim, Lawrence C. Layman, Hyung-Goo Kim

**Affiliations:** Hamad Bin Khalifa University; RIKEN BioResource Research Center; Hamad Bin Khalifa University; Laboratory Corporation of America Holdings; Harvard Medical School; Hospital Santa Maria, Centro Hospitalar Universitário Lisboa Norte; University of Newcastle; Forster Genetics-Hunter New England Local Health District; CHU de Caen Normandie; CHU de Caen Normandie; University of Amsterdam; Louisiana State University; University of London; Kent State University; Harvard Medical School; Harvard Medical School; Chungnam National University; Augusta University; Hamad Bin Khalifa University

## Abstract

In an apparently balanced translocation t(7;12)(q22;q24)dn exhibiting both Kallmann syndrome (KS) and intellectual disability (ID), we detected a cryptic heterozygous 4.7 Mb del(12)(p11.21p11.23) unrelated to the translocation breakpoint. This new finding raised the possibility that KS combined with neurological disorder in this patient could be caused by gene(s) within this deletion at 12p11.21–12p11.23 instead of disrupted or dysregulated genes at the genomic breakpoints. Screening of five candidate genes at both breakpoints in 48 KS patients we recruited found no mutation, corroborating our supposition. To substantiate this hypothesis further, we recruited six additional subjects with small CNVs and analyzed eight individuals carrying small CNVs in this region from DECIPHER to dissect 12p11.21–12p11.23. We used multiple complementary approaches including a phenotypic-genotypic comparison of reported cases, a review of knockout animal models recapitulating the human phenotypes, and analyses of reported variants in the interacting genes with corresponding phenotypes. The results identified one potential KS candidate gene (*TSPAN11*), seven candidate genes for the neurodevelopmental disorder (*TM7SF3, STK38L, ARNTL2, ERGIC2, TMTC1, DENND5B*, and *ETFBKMT)*, and four candidate genes for KS with ID (*INTS13, REP15, PPFIBP1*, and *FAR2)*. The high-level expression pattern in the relevant human tissues further suggested the candidacy of these genes. We propose that the dosage alterations of the candidate genes may contribute to sexual and/or cognitive impairment in patients with KS and/or ID. Further identification of point mutations through next generation sequencing will be necessary to confirm their causal roles.

## Introduction

Kallmann syndrome (KS) is a clinically and genetically heterogeneous disorder characterized mainly by the co-occurrence of idiopathic hypogonadotropic hypogonadism (IHH) due to the defective action of hypothalamic gonadotropin releasing hormone (GnRH) through the hypothalamic-pituitary-gonadal axis, and anosmia associated with dys/agenesis of the olfactory bulbs. If untreated with GnRH, gonadotropins or sex steroids, most patients show a delayed or absence of pubertal development, which results in the impairment of reproductive function. Its prevalence has been estimated to be 1/10,000 in males and 1/50,000 in females. To date, at least 44 genes have been implicated in the cause of KS/IHH^1^. Four chromosomal rearrangements involving the chromosome 12q24 region were reported in patients with KS and hypogonadism. Three of them are balanced chromosomal translocations associated with a) KS in a patient with t(7;12)(q22;q24)dn^2^ b) IHH in a patient with t(4;12)(q25;q24.2)dn^3^, c) severe primary hypogonadism in three brothers with t(1;12)(p32;q24)^4^. Lastly, a del(12)(q24.31q24.33) is involved in IHH^5^. Molecular characterization of breakpoints in balanced chromosomal rearrangements associated with an abnormal phenotype has been instrumental in positional cloning of disease genes^6–9^. In KS, *FGFR1* and *WDR11* were identified by positional cloning of balanced translocations^10,11^. Since four reported chromosomal rearrangements^2–5^ suggest the presence of a putative KS gene at the overlapping region of 12q24, we acquired the available lymphoblastoid cell lines of Subject 1 with a *de novo* apparently balanced translocation t(7;12)(q22;q24)^2^ from the Coriell Institute for Medical Research (www.coriell.org)^12^. By positional cloning, we mapped and cloned both breakpoints, and this revealed a non-coding RNA, *RMST*, directly truncated at the chromosome 12 breakpoint^13^. This result was recapitulated by targeted breakpoint sequencing^9^ and genome sequencing^14^ recently. Further sequencing of five genes located at or near the breakpoints on both chromosomes revealed no pathogenic variants in 48 KS patients we recruited. Although the chromosome translocation in this patient appeared balanced, a subsequent microarray detected a cryptic 4.7 Mb heterozygous microdeletion at 12p11.21–12p11.23, which was not reported and will likely contain the genomic loci of candidate gene(s) for KS and ID in Subject 1. *In silico* genomic analysis, using 15 CNVs within this genomic region suggested one KS candidate gene, seven ID candidate genes, and four candidate genes for KS combined with ID.

By taking advantage of our candidate genes, the isolation of pathogenic heterozygous variants will be accelerated through mining of next generation sequencing (NGS) databases of individuals with delayed or absent pubertal development. In addition, given the extensive phenotypic heterogeneity of mental disorders, our candidate genes in this genomic region will further help identify disease-associated variants from VUS (variants of unknown significance) databases of individuals with neurodevelopmental disorders including autism.

## Results

**Figure 1:**
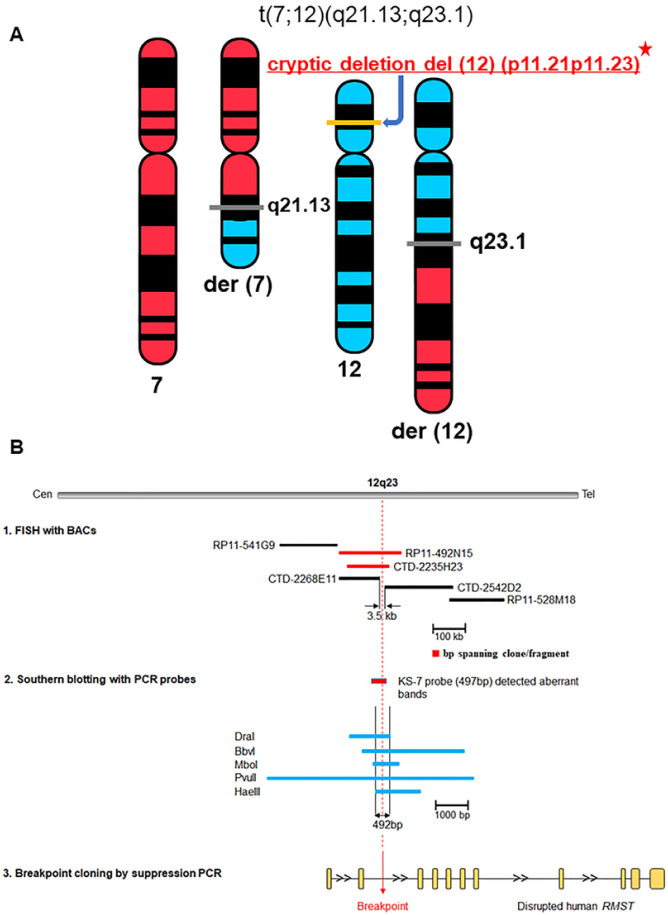
(A) Ideogram illustrating the revised t(7;12)(q21.13;q23.1)dn karyotype in subject 1, DGAP032. After breakage of two chromosomes, the reciprocal exchange of chromosome segments between chromosomes 7 and 12 has taken place, generating two derivative chromosomes in the subject with two horizontal gray bars at the breakpoint positions. On chromosome 12, the deleted cryptic segment at 12p11.21–12p11.23 identified was depicted as a horizontal yellow bar. (B) Physical mapping of the 12q23 translocation breakpoint of DGAP032 by FISH and Southern blot hybridization. Diagram shows breakpoint refined by FISH and Southern blot. For FISH, two BAC clones RP11–492N15 and CTD-2235H23 spanning the breakpoint, which is represented as a dashed vertical red line, were identified, and shown as red bars. The breakpoint was further narrowed to 3.5 kb between CTD-2268E11 and CTD-2542D2 shown as black bars. Southern blot analysis using Blot 4 with the probe KS-7 identified aberrant fragments of subject DNA digested with five different restriction enzymes (DraI, BbVI, MboI, PvuII, and HaeIII, Figure 3B). The breakpoint was refined to 492 bp between the centromeric end of HaeIII and the telomeric end of DraI, which was then isolated with suppression PCR and sequenced. The breakpoint at 12q23.1 is located in intron 2 of *RMST* (NR_152618.1).

Positional cloning was undertaken to clone the translocation breakpoints and to identify a gene possibly affected by the apparently balanced translocation in Subject 1, who was published with karyotype and clinical information (Fig. 1A)^2^. By constructing a bacterial artificial chromosome (BAC) contig covering the breakpoint, we mapped and narrowed the translocation breakpoints by fluorescence *in situ* hybridization (FISH) and Southern blot analysis. Cloning of the genomic breakpoint on chromosome 12 by suppression PCR led to identification of the non-coding RNA *RMST* (Rhabdomyosarcoma 2 Associated Transcript, MIM 607045), which was directly disrupted by the breakpoint at 12q23.1 (Fig. 1B). The breakpoint on chromosome 7 was localized at 7q21.13, 37 kb upstream of the predicted gene *ZNF804B* (Zinc Finger Protein 804B) with an unknown function. This breakpoint mapping resulted in the revision of the karyotype to t(7;12)(q21.13;q23.1) *dn* (Fig. 1A)^13^. When we first conducted positional cloning between 2002 and 2008^13^, we initially considered that *RMST* might represent a potential candidate gene due to two other cases involving chromosome 12q24 with IHH. Nevertheless, our screening of five genes including *RMST* at, and around, both breakpoints in 48 KS patients did not identify any pathogenic variant. Therefore, the translocation breakpoint at 12q23 could not harbor a positional candidate gene for KS.

**Figure 2:**
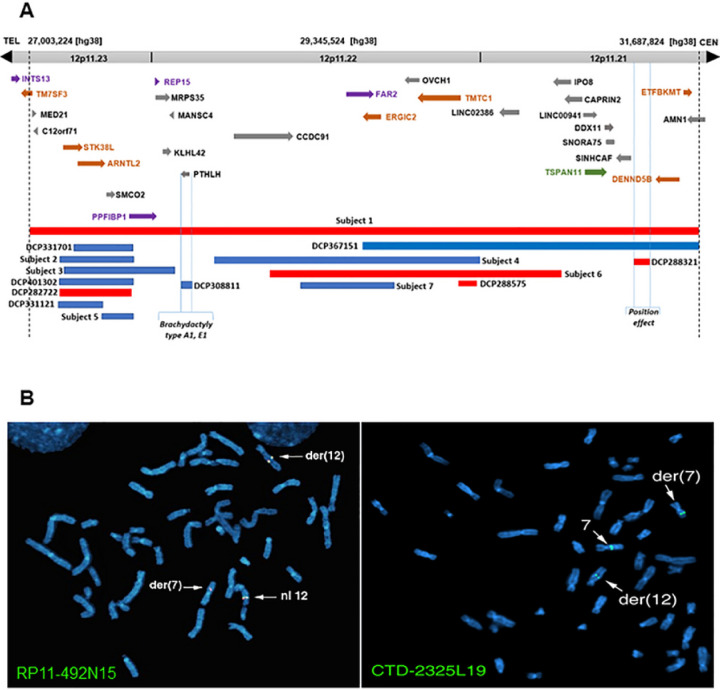
(A) Cryptic 4.7 Mb heterozygous deletion encompassing 29 genes located from 12p11.21 to 12p11.23. Eight heterozygous CNV cases from the DECIPHER database are denoted by DCP along with six heterozygous CNVs (subjects 2–7) we recruited. These 14 CNVs are encompassed in subject 1 to help narrow down the candidate gene region by *in silico* comparative genomic mapping. Red bars represent deletions, whereas blue bars represent duplications. One gene in green is a candidate for Kallmann syndrome, whereas seven genes in brown are NDD candidates. Four genes in purple are chosen as candidates for KS combined with ID. Arrow indicates transcriptional direction of each gene. (B) FISH with two BAC clones spanning the breakpoints at 12q23.1 and 7q21.13, respectively. BAC clone RP11–492N15 shows normal signals on normal chromosome 12 and split signals on both derivative chromosomes 7 and 12. (2). BAC clone CTD-2325L19 with normal signals on normal chromosome 7 and split signals on both derivative chromosomes 7 and 12.

aCGH with increased resolution can reveal CNVs (copy number variations) that remain hidden from karyotyping^15^. Because cryptic deletions are a common finding in seemingly balanced translocations^16,17^, we performed aCGH which revealed a heterozygous 4.7 Mb deletion encompassing 29 known and predicted genes at 12p11.21–12p11.23 (Fig. 2A). Previous chromosome analysis could not have detected this cryptic heterozygous deletion in this patient with an apparently balanced translocation, because the resolution of karyotyping was limited to around 5 Mb or higher^18^. To identify the positional candidate gene(s) for KS and/or intellectual disability, we have recently recruited additional six patients with microdeletions and microduplications (Subjects 2–7) at 12p11.21–12p11.23 (Table 1 and Fig. 2A). Importantly, these seven subjects share an overlapping phenotype of developmental delay (DD), intellectual disability (ID), learning disability, and language/speech delay. Some have autism, craniofacial anomalies (CFA), and epilepsy (Table 1). This suggests the presence of genomic loci of neurodevelopmental genes within this region. *In-silico* comparative mapping of these seven subjects with eight informative CNVs (https://decipher.sanger.ac.uk/, version 11.14)^19^ comprising five microdeletions and ten microduplications within the microdeletion in Subject 1, has allowed us to implicate one KS candidate gene, seven neurodevelopmental candidate genes, and four candidate genes for KS coupled with neurodevelopmental disorders.

### Breakpoint region was refined to 3.5 kb at 12q23.1, and 87 kb at 7q21.13 by FISH mapping

Breakpoint mapping by FISH was initiated with clones RP11–1103 and RP11–77E2 on 7q21 and clones RP11–74K11 and RP11–1K22 on 12q23. These clones flanked the breakpoints, and subsequent experiments were performed until breakpoints containing clones were identified as described^8^. In final experiments mapping the chromosome 12 breakpoint, clones RP11–492N15 and CTD-2235H23 hybridized to chromosome 12, der(12) chromosome, and der(7) chromosome, indicating that the translocation breakpoint of chromosome 12 is located within the sequence of these two BAC clones (Fig. 1B). Figure 2B illustrates hybridization of SpectrumGreen labeled RP11–492N15 to chromosome 12 as well as both derivative chromosomes. CTD-2542D2 hybridized to chromosome 12 and der(7) chromosome, whereas CTD-2268E11 hybridized to chromosome 12 and der(12) chromosome, demarcating the breakpoint region at 12q23 (Fig. 1B).

**Figure 3:**
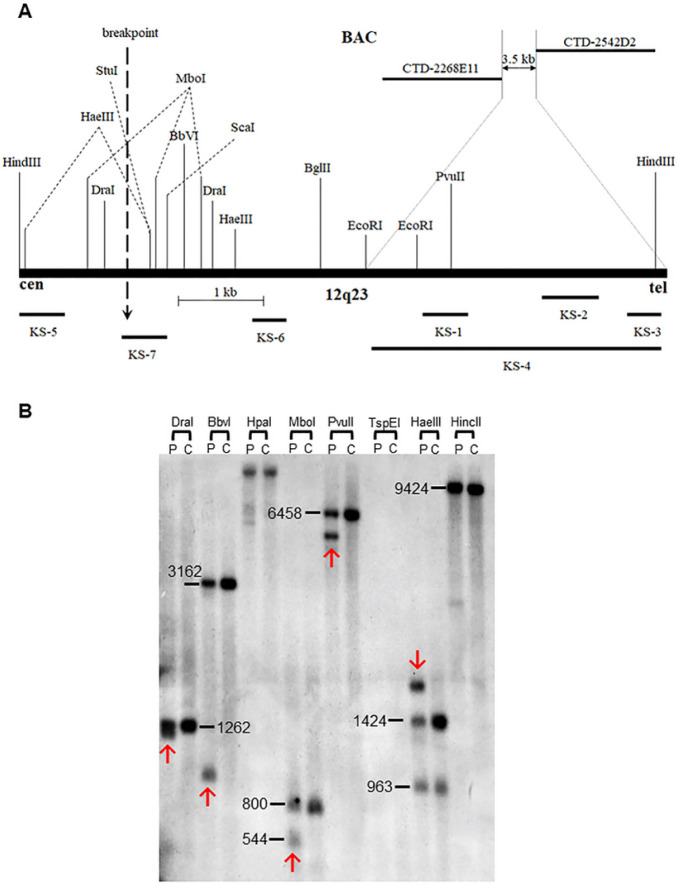
(A) Restriction map of a 7.3 kb HindIII genomic fragment (97,460,797–97,468,119 / hg38) containing the breakpoint on 12q23. BamHI, HincII, HpaI and SnaBI do not have restriction sites on this map and only the relevant restriction sites in relation to the fragments detected by shown probes are indicated. Positions of the PCR-derived probes KS-1 to KS-7 used for the breakpoint mapping by Southern analysis are indicated below. Note that the breakpoint is located 2.7 kb upstream of the region narrowed by FISH. (B) Genomic DNA blots hybridized with probes from the 12q23 breakpoint region. Each lane contains genomic DNA digested with the designated restriction enzymes from either DGAP032 (P) or a normal control (C). Additional bands in the P lanes indicate novel restriction junction fragments generated by the interchromosomal exchange. The hybridization probe KS-7 detected aberrant bands indicated by red arrows containing breakpoints at 12q23. The numbers next to normal restriction fragment seen in both subject and control lanes indicate the size of genomic restriction fragment in bp. Full original image is shown here without any cropping.

These results indicate that the chromosome 12 breakpoint was within or adjacent to a 3.5 kb interval (chrl2: 97,460,688 – 97,464,146 / hg38) between CTD-2542D2 and CTD-2268E11 based on the placement of end sequenced BAC clones on the current genomic sequence map (Fig. 1B and Fig. 3A). This interval was 874 bp upstream from the 5’ end of the *RMST* locus.

The chromosome 7 breakpoint was mapped within clone CTD-2325L19 (Fig. 2B). Clone RP11–46013 was telomeric to the breakpoint. Based on the locations of these end-sequenced clones, the chromosome 7 breakpoint is located within an 87 kb interval (data not shown). This interval does not contain any genes and the next nearest gene, *ZNF804B*, was approximately 11 kb beyond the telomeric boundary of the chromosome 7 breakpoint region defined by FISH mapping.

#### Breakpoint region was refined to 492 bp at 12q23.1 by Southern blot analysis

To map the breakpoints, at which the disease gene might be disrupted or dysregulated in the Subject 1, Southern blot analysis was performed from the breakpoint region on chromosome 12, which was refined by FISH. Probes KS-1, KS-2, and KS-3 were hybridized sequentially to a filter (Blot 1) containing genomic DNAs of the translocation subject and normal controls digested by BglII, DraI, EcoRI, EcoRV, HaeIII, HindIII, RsaI, and ScaI. All three probes detected the same rearrangement seen in a 7.3 kb genomic HindIII fragment in the subject, which was not observed in controls (data not shown). Thus, the breakpoint is located within a 7.3 kb HindIII restriction fragment (chr12: 97,460,797 − 97,468,119 / hg38), which includes 3,350 bp of the 3.5 kb putative breakpoint region narrowed by FISH (Fig. 3A). To exclude the possibility that this aberrant band did not come from a polymorphism, the probe KS-4 was hybridized with nylon membrane Blot 2 with genomic DNAs digested by BamHI, StuI, HincII, NaeI, Sau3AI, SfoI, SnaBI, and SspI. One aberrant band was detected in the subject’s genomic DNA lane with SnaBI beneath the 12.3 kb control SnaBI restriction fragment, which includes the putative 7.3 kb breakpoint region narrowed by the HindIII restriction fragment. This confirms that the aberrant band detected by HindIII is caused by chromosome rearrangement (data not shown).

Because the putative breakpoint region defined by the HindIII rearranged fragment included a new 3.8 kb region, probes KS-5 and KS-6 were hybridized respectively to the genomic DNAs from the patient and normal control digested by BamHI, StuI, HincII, DraI, EcoRI, HindIII, RsaI, and SspI on two nylon membranes of Blot 3. The probe KS-5 detected rearranged fragments in the patient genomic DNAs digested with BamHI, StuI, HincII, EcoRI, and HindIII, whereas the probe KS-6 detected rearranged fragments on the subject lanes with EcoRI and HindIII (data not shown). The breakpoint region located within a 3.9 kb StuI restriction fragment (chr12: 97,466,625 – 97,470,522 / hg38) overlaps with the breakpoint region of 7.3 kb HindIII restriction fragment, narrowing the breakpoint to 1.5 kb (chr12: 97,466,625 – 97,468,119 / hg38).

To confirm and further narrow the 1.5 kb breakpoint region, the probe KS-7 was hybridized respectively with one nylon membrane (Blot 4) containing digested genomic DNAs with DraI, BbvI, HpaI, MboI, PvuII, HaeIII, and HincII. Rearranged bands were detected on the subject DNA lanes with DraI, BbvI, MboI, PvuII, HaeIII, and HincII on the first membrane (Fig. 3B), narrowing the breakpoint to a 492 bp region (chr12: 97,466,625 – 97,467,118 / hg38, Fig. 1B).

### Breakpoint cloning of t(7;12)(q21.13;q23.1) identified RMST truncated at 12q23.1

#### Cloning of the breakpoint from derivative chromosome 12

**Figure 4:**
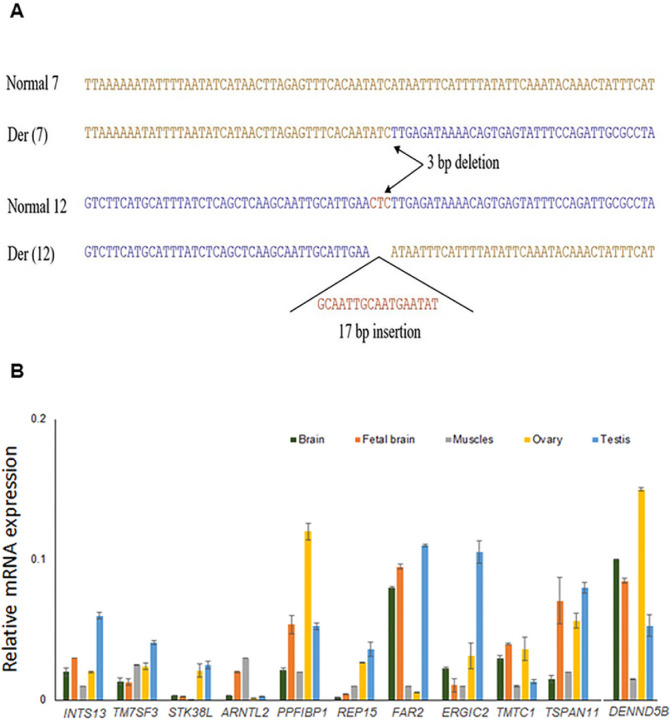
(A) Sequences of the junction fragment composed of two different chromosomes at the translocation breakpoints. Sequence comparison of the normal chromosomes 7 and 12 with der(7) and der(12) at the breakpoints junctions. Three bp sequence CTC from chr12 is deleted at the junction of der(7), and a 17 bp insertion was found at the junction of the der(12). (B) Transcript levels of *INTS13, TM7SF3, STK38L, ARNTL2, PPFIBP1, REP15, FAR2, ERGIC2, TMTC1, TSPAN11*, and *DENND5B* in five different human tissues (i.e., brain, fetal brain, muscle, ovary and testis) were determined by RT-qPCR. Varying levels of expression of these candidate genes were detected in different tissue samples.

The 3.5 kb junction fragment detected on Blot 3 with probes KS-5 and KS-6 from EcoRI digestion from derivative chromosome 12 was amplified by two independent suppression PCRs^20^ using the primers sets under the condition mentioned in Materials & Methods section. The size of these two PCR products were 0.6 and 0.8 kb respectively, and sequence analysis of these fragments confirmed that they were the junction fragments from the der(12). The genomic breakpoint was located between 97,466,873 and 97,466,877 (hg38) at 12q23.1 (Fig. 4A), within the second intron of RMST (Fig. 1B). As this junction fragment contained chromosome 7 sequences adjacent to the breakpoint, a BAC CTD-2325L19 was identified from this sequence by BLAT at Human Genome Browser (hg38), which mapped to 7q21 and was contained in the 3.5 Kb narrowed region by FISH. BAC clone CTD-2325L19 also contained chromosome 7 sequence of this junction fragment and showed hybridization signals of SpectrumGreen on chr7, der(7), and der(12) from FISH (Fig. 2B, right picture). This result indicated that BAC clone CTD-2325L19 contained the 7q21 breakpoint of the subject.

#### Cloning of the breakpoint from derivative chromosome 7

Because the sequence of chromosome 7 adjacent to the breakpoint of der(12) was now known, the 2.3 kb junction fragment from der(7) was generated by nested PCR using primers proximal to the 7q21.13 breakpoint and ones distal to the 12q23.1 breakpoint using the primers sets under the condition mentioned in Materials & Methods section: Sequence analysis confirmed that this fragment was the junction fragment from der(7). The genomic breakpoint was located between 88,722,752 and 88,722,753 (hg38) at 7q21.13 (Fig. 4A). Two junction fragment sequences were evaluated to determine if there had been any gain or loss of chromosome material at the site of the translocation. Comparison of the normal chromosome sequence at 7q21.13 and normal chromosome sequence at 12q23.1 along with those from the two junction fragments revealed that there had been an unknown 17 bp insertion (GCAATTGCAATGAATAT) in the der(12) junction fragment and 3 bp CTC deletion from chromosome 12 from the der(7) junction fragment^13^ (Fig. 4A).

#### Identification of three candidate genes at 12q23.1 from t(7;12) (q21.13;q23.1)

We mapped and sequenced both translocation breakpoints to identify a gene that underlies KS. Because two balanced translocations and one deletion 12q24 involving hypogonadism were published^3–5^, sequences upstream and downstream of the chromosome 12 breakpoint were analyzed for the existence of a candidate gene, which resulted in the identification of *RMST*. By comparison of the genomic sequence, it was determined that the translocation directly disrupted *RMST* such that the 12q23 breakpoint was located within intron 2, downstream of the second exon of this gene (Fig. 1B)^13^. *RMST* encodes a long non-coding RNA specifically expressed in the developing brain.

Another candidate gene, *NEDD1* (Neural Precursor Cell Expressed, developmentally down regulated, MIM 600372), was mapped 513 kb upstream from the breakpoint, whereas pseudogene *PAFAH1B2P2* (PAFAH1B2 pseudogene 2) was mapped 248 kb downstream from the breakpoint.

#### Identification of two candidate genes at 7q21.13 from t(7;12) (q21.13;q23.1)

In a *de novo* balanced translocation with associated phenotype, the culprit gene is often located at one of two breakpoints^7,8,10,11^. We have looked at genes located at chromosome 7 breakpoint too. The closest gene to the breakpoint at 7q21.13 is *ZNF804B*, which was mapped 37 kb distal, whereas *STEAP4* (Six-Transmembrane Epithelial Antigen of Prostate 4 aka STEAP4 Metalloreductase, MIM 611098) was mapped 416 kb proximal to the breakpoint^13^.

#### Identification of the autosomal dominant candidate genes for KS and/or ID from del(12)(p11.21p11.23)

Array CGH analysis revealed a heterozygous 4.7 Mb interstitial microdeletion (Chr12: 27,003,224 − 31,687,824 /hg 38) in the chromosomal band at 12p11.21–12p11.23 (Fig. 1A and Fig. 2A). Based on the aberrant karyotype and aCGH results found in Subject 1, the nomenclature is revised as 46,XY,t(7;12)(q21.13;q23.1)*dn*, arr[hg 38] 12p11.23p11.21(27,003,224 − 31,687,824)x1 (Fig. 1A), which was reported back to Coriell.

By *in silico* comparative genomic mapping^21–25^ at 12p11.21–12p11.23, we have identified a total of 12 autosomal dominant positional candidate genes for KS and/or ID. They include one candidate gene for KS, *TSPAN11*, seven ID candidate genes, *TM7SF3*, *STK38L*, *ARNTL2*, *ERGIC2*, *TMTC1*, *DENND5B*, *ETFBKMT*, and four candidate genes for KS coupled with ID, *INTS13*, *PPFIBP1*, *REP15*, and *FAR2* (Fig. 2A and Table 2).

#### Validation of putative candidate genes using tissue-specific RT-qPCR

To investigate the functional significance of 11 positional candidate genes among 12 in phenotype-relevant tissues, RT-qPCR transcript levels were measured in five distinct human tissues (brain, fetal brain, muscles, ovary, and testis). The spatiotemporal regulation of gene expression causes varying expression patterns, which also depend on a number of variables, including the RNA isolation process and detection techniques. We used commercially available human RNA samples to measure the expression patterns through RT-qPCR experiments so that we would have a reference of the expression of the genes of our interest. This was done because different expression patterns were found in several publicly available resources, including the GTEx Portal (https://gtexportal.org/home/) and NCBI (https://www.ncbi.nlm.nih.gov/).

Our KS-candidate gene *TSPAN11* showed high expression in testis and ovary, whereas five NDD-candidate genes (*TM7SF3, ARNTL2, ERGIC2, TMTC1*, and *DENND5B*) among 7 showed good expression levels in brain and fetal brain. Our four candidate genes for KS + NDD- *INTS13, PPFIBP1, REP15*, and *FAR2* showed expression in ovary and testis along with adult and fetal brain tissues (Fig. 4B). The expression of these genes in tissue samples relevant to the disease-causing organs suggests that they might underlie the clinical phenotype when mutated. The NDD candidate gene *ETFBKMT* was not included in this experiment, because it is identified as an additional ID candidate gene after re-interrogation of all 29 genes at 12p11.21–12p11.23.

## Discussion

So far, three balanced translocations and one microdeletion with hypogonadism or KS involving 12q24 have been reported^2–5^. In one case reported in 1983, all three brothers of Vietnamese Chinese origin were revealed to have severe primary hypogonadism with 46,XY,t(1;12)(p32;q24)^4^. In the second case, an apparently balanced translocation t(7;12)(q22;q24) in a male with KS and intellectual disability was published in 1990^2^. Another case reported a balanced chromosomal translocation between the distal q arms of chromosome 4 and 12, t(4;12)(q25;q24.2), in a Turkish male patient in 1994, who showed IHH and a lack of secondary sexual characteristics yet had a normal sense of smell^3^. After the three aforementioned balanced translocations with hypogonadism were published, a *de novo* interstitial deletion del(12)(q24.3q24.33) was described in a male with ambiguous genitalia and development delay in 1999^5^.

We postulated that all four reported chromosomal rearrangements involving the 12q24 region could be explained by the haploinsufficiency of a gene responsible for KS or IHH. The positional cloning of balanced translocation patients was successful to identify KS genes at the translocation breakpoints^10,11^. To investigate if the translocation might contribute to the phenotypes in the male Subject 1, we mapped and sequenced both translocation breakpoints to clone genes that may be involved in KS seen in this individual with t(7;12)(q22;q24)^13^.

Physical mapping of the translocation breakpoint by FISH and Southern blot hybridization led to the identification of five genes at both breakpoints. *ZNF804B* mapped 37 kb distal from the breakpoint is the closest gene to the breakpoint at 7q21.13^9^ and *STEAP4* mapped 416 kb proximal to the breakpoint. The breakpoint at 12q23.1 directly disrupts the non-coding RNA *RMST*, whereas *NEDD1* and *PAFAH1B2P2* map closest to the breakpoint on the proximal and distal sides, respectively. Based on the molecular analysis results, the locations of the cytogenetic bands on both chromosomes 7 and 12 of the apparently balanced chromosome translocation have been precisely revised as t(7;12)(q21.13;q23.1)^9,13,14^. We assumed that the causative gene for KS is located on chromosome 12 due to two previously reported chromosomal rearrangements displaying overlapping phenotypes of KS and screened for mutations in three-selected candidate genes (*RMST, NEDD1, PAFAH1B2P2*) based on their proximity to the 12q23.1 breakpoint. *RMST* is disrupted in intron 2, and mutation screening of this gene was performed in 48 KS subjects negative to *ANOS1* and *FGFR1*. We identified a heterozygous nucleotide change in *RMST* (heterozygous change of C/C to C/T at the 214th nucleotide in exon 10 of *RMST* NR_152618.1) in a KS male alongside his mother with anosmia. The nucleotide change turned out to be a polymorphism, as two healthy sisters of the patient shared the same nucleotide change. Additionally, the two patients in this family were found to have a mutation of *FGFR1* (c.821G > A, p.E274G)^26^, suggesting this gene as the cause of the phenotype in this family. *PAFAH1B2P2* and *NEDD1*, mapping 248 kb downstream and 513 kb upstream from the breakpoint, respectively, were also screened in the same collection of 48 KS patients we recruited, with no evidence of mutations.

Two additional genes were mapped in the breakpoint region at 7q21.13. *ZNF804B*, which has an unknown function, is located 37 kb downstream from the breakpoint; hence, it is the closest gene to the breakpoint^9^. ZNF804B is a member of the zinc finger protein family, which is composed of four exons. *ZNF804B* has not previously been described as a disease-related gene with substantial evidence. There is limited information in the literature regarding its full biological functions, except it was found to be one of the candidate genes associated with autism spectrum disorder and neurodevelopmental disorders^27,28^. This suggests that *ZNF804B* might be dysregulated by position effect and could be the cause of intellectual disability of Subject 1. In addition, *STEAP4* at 7q21.12 is located 416 kb upstream from the breakpoint. Functioning as metalloreductase, *STEAP4* resides in the Golgi apparatus and may be involved in adipocyte development and metabolism. Mutation screening of both genes in the same cohort of 48 patients we recruited did not detect any putative mutations, though polymorphisms were identified. Additionally, we screened for mutations in *ANOS1* and *FGFR1* in the t(7;12) translocation subject. No potential disease-causing mutations were found (data not shown). We cannot exclude the possibility of mutation of other causative gene(s) for KS in this subject.

Six percent of carriers of balanced translocations manifest abnormal phenotypes^29^ due to the disruption of the genes at the breakpoints or dysregulation (*i.e*., position effect) resulting in reduced expression of the genes by the separation from its cis regulatory elements^30^. Nonetheless, 40% of the patients with apparent balanced translocations were reported to have at least one deletion at one of the breakpoints or genomic regions elsewhere, suggesting that deletions might be common in apparently balanced chromosome rearrangements^16^.

As we did not find any mutations in five candidate KS genes at or near both breakpoints from the apparent balanced translocation, we performed aCGH analysis, which remarkably unmasked a cryptic 4.7 Mb submicroscopic microdeletion at 12p11.21–12p11.23. This cryptic microdeletion was not detected in previous studies of the breakpoints of Subject 1 (DGAP032) on the molecular level^9,13,14^.

There has been a report of chromosomal variation including CNVs in lymphoblastoid cell lines (LCLs). In their study, Shirley et al found more CNVs per sample on average for LCLs than for PBMCs, but the differences were statistically not significant. Furthermore, chromosomal regions associated with a significantly different number of CNVs did not include chromosome 12^31^.

Thus, it is unlikely that del(12)(p11.21p11.23) is a culture induced artifact, although we cannot exclude this possibility entirely. Unfortunately, the *de novo* status of this microdeletion could not be determined due to the unavailability of parental samples.

In a patient with two different concomitant genomic rearrangements such as an unbalanced translocation and a simultaneous translocation-unrelated microdeletion or microduplication, the culprit gene was found to be located at one translocation genomic breakpoint sometimes albeit rare. For instance, in a female subject affected with autism and ID with t(14;21)(q21.1;p11.2)*dn* and 2.6 Mb of microdeletion comprising 15 genes at 2q31.1, the causative gene *LRFN5* (Leucine-Rich Repeat and Fibronectin Type III Domain-Containing Protein 5, MIM 612811) was found dysregulated at the 14q21.1 translocation breakpoint^32^. In majority of cases, however, the disease gene is within CNV as exemplified in two positional ID candidate genes- *VAMP8* (Vesicle-Associated Membrane Protein 8, MIM 603177) and *RNF181* (Ring Finger Protein 181, MIM 612490) - identified in a cryptic 390 kb duplication region in a subject with unbalanced t(8;10)(p23.3;q23.2)^21^.

The result of aCGH in Subject 1 raised a new possibility that the heterozygous 4.7 Mb interstitial deletion containing 29 genes at 12p11.21–12p11.23 (chr12: 27,003,224 – 31,687,824 /hg 38) (Fig. 1A and Fig. 2A) instead of the genomic breakpoints of the reciprocal translocation might harbor a KS gene in this subject. This hypothesis is corroborated by DECIPHER case 284660 (not listed in Fig. 2A), with a 7.09 Mb heterozygous deletion (chr12:22,444,774 – 29,533,886 [hg38]) exhibiting cryptorchidism and mild global developmental delay (https://decipher.sanger.ac.uk/). This microdeletion overlaps a 2.53 Mb genomic region with our subject with KS (chr12:27,003,224 – 29,533,886 [hg38]).

In addition to KS, Subject 1 with an unbalanced chromosome translocation presented with ID^2^. Although the genes causing IHH associated with ID have been recently reported^33,34^, more than one genes contributing to the comprehensive phenotype are alternative explanations in contiguous gene deletion syndromes represented by Potocki-Shaffer-Syndrome^7^ or a deletion on chromosome X causing KS coupled with ID^35^.

This microdeletion encompassing 29 genes is likely to harbor the disease genes involved in the common set of neurodevelopmental phenotypes shared with our seven studied CNV patients and eight unpublished CNV cases from the DECIPHER database (Fig. 2A, Tables 1 and 3).

Among 15 heterozygous CNVs at 12p11.2, duplications account for ten cases, whereas deletions represent the remaining five cases (Fig. 2A, Tables 1 and 3). At least one neurodevelopmental phenotype was seen in each case. Some of the CNVs were inherited from one parent with an unknown phenotype, whereas the inheritance of the remainders is unknown (Tables 1 and 3). This suggests that some genes in the CNVs located at 12p11.2 may have incomplete penetrance or epigenetic imprinting if a carrier parent is asymptomatic. We interpret the pathogenicity of deletion and duplication cases similarly since they both exhibit the similar neurological condition. This is because any CNV might interfere with the strict stochiometric control of genes on a protein expression level^36^.

Based on *in silico* comparative genomic analysis at 12p11.21–12p11.23, we suggest one putative KS candidate gene, *TSPAN11* (Tetraspanin 11). One missense variant c.203G > A (NM_001080509.3) leading to an amino acid substitution Glycine to Aspartic acid at position 68 was reported in a KS patient^37^. This variant shows high deleterious CADD score of 25.8 (HG38).

Furthermore, seven intellectual disability candidate genes, *TM7SF3, STK38L, ARNTL2, ERGIC2, TMTC1, DENND5B* and *ETFBKMT* have been identified (Table 2).

Putative position effect of *TM7SF3* and the inclusion of *STK38L* and *ARNTL2* in CNVs at 12p11.23 along with their sporadic variants reported in neurodevelopmental disorder (NDD) patients will likely explain their candidacy. A de novo missense variant in *TM7SF3* (Transmembrane 7 Superfamily Member 3, MIM 605181) was identified in a patient with a neurodevelopmental disorder^28,38^. One missense variant in *HNRNPL* (Heterogeneous Nuclear Riboprotein L, MIM 603083), an interacting protein of *TM7SF3*^39^, was described in an ID patient^40^.

*STK38L* (Serine/Threonine Kinase 38 Like, aka NDR2, Nuclear Dbf2 Related Kinase 2, MIM 615836) regulates the morphology and division of neuronal cells^41–43^, and integrin-dependent dendritic and axonal growth in mouse hippocampal neurons^44^.

Consequently, Stk38l KO mice exhibit arbor-specific alterations of dendritic complexity in the hippocampus^44^. *De novo* nonsense and missense variants in *STK38L* have been identified in individuals with autism spectrum disorder^45^ and schizophrenia^46^, respectively. Genes mutated in schizophrenia are also mutated in autism and ID^46^.

The third candidate gene of ID at 12p11.23 is *ARNTL2* (Aryl Hydrocarbon Receptor Nuclear Translocator-Like Protein 2, MIM 614517), and its missense and nonsense variants were reported in patients with autism^47^, and in developmental and epileptic encephalopathy^48^, respectively. Proteins that physically interact with one another frequently participate in the same biological activity, and mutations in these genes may result in similar clinical features. Among the interactors of *ARNTL2, CTTNBP2* (Cortactin Binding Protein 2, MIM 609772)^49^ with 26 de novo genetic variants were identified in probands with autism/development delay^50^. Another interactor *UBE3A* (Ubiquitin-Protein Ligase E3A, MIM 601623)^51^ is a well-known Angelman syndrome gene^52^ and its two frameshift variants were also reported in autistic individuals^53,54^. Eight variants in *PER2* (Period Circadian Regulator 2, MIM 603426), another interactor^55^, were described in individuals with autism^45,56,57^.

Two ID candidate genes, *ERGIC2* and *TMTC1*, were found at 12p11.22. One frameshift variant in *ERGIC2* (Endoplasmic Reticulum-Golgi Intermediate Compartment Protein 2, MIM 612236) has been reported in an individual with ASD^58^. ERGIC2 physically interacts with SLC39A8 (Solute Carrier Family 39, Member 8, MIM 608732)^55,59^ and CUX1 (Cut-Like Homeobox, 116896)^60^, which are associated with autosomal recessive syndromic intellectual disability^61^ and non-syndromic intellectual disability/development delay^62^, respectively. As interactors of ERGIC2, two catalytic subunits of Rab GTPase activating proteins RAB3GAP1 (RAB3 GTPase-Activating Protein, Catalytic Subunit, 602536)^60^ and RAB3GAP2 (RAB3 GTPase-Activating Protein, Noncatalytic Subunit, MIM 609275)^60^ cause autosomal recessive Warburg Micro syndrome including developmental abnormality of the central nervous system, when mutated homozygously^63–65^.

In addition, a *de novo* missense variant in *TMTC1* (TransMembrane and Tetratricopeptide repeat Containing 1, MIM 615855) was found in a child with neurodevelopmental disorder^28^. The Tetratricopeptide repeat (TPR) structural motif contained in this gene is reported to present as a functional domain in *NAA15*^66^, *OGT*^67–69^, *TANC2*^70^, and *TTC25*^71^, all of which are associated with autism and intellectual disability. TMTC1 interacts with BCOR (BCL6 Corepressor, 300485)^72^ and VIRMA (Vir like M6A Methyltransferase Associated, MIM 616447) (aka KIAA1429)^73^. The mutations of the former cause Lenz microphthalmia, an X-linked syndromic intellectual disability^74^; however, the variants of the latter were found in individuals with ASD^45^, developmental delay^28^, schizophrenia^45,46^, and Tourette syndrome^75^.

At 12p11.21, we identified two additional ID candidate genes. One ID candidate gene is *DENND5B* (DENN Domain Containing 5B, MIM 617279), a guanine nucleotide exchange factor (GEF) mediating the activation of small GTPases. With many downstream targets, they function as molecular switches in intracellular signaling pathways. There are some known GEFs involved in NDDs. Apart from *IQSEC2* associated with X-linked intellectual disability, many variants in GEFs *HERC1*^27,76^, *TRIO*^77,78^, *ARHGEF9*^79,80^, and *ARHGEF10*^81^ have been reported in patients with intellectual disability, epilepsy, and/or autism. *VAV3* (VAV Guanine Nucleotide Exchange Factor 3, MIM 605541) identified as an NDD candidate gene at 1p13.3, because of the KO mouse phenotype, its genomic position, and reported variants, is a GEF^82^. Variants in *RAB11A* (RAS-Associated Protein, MIM 605570)^83^ and *GRB10* (Growth Factor Receptor-Bound Protein 10, MIM 601523)^84^, interactors of *DENND5B*, are found in individuals with developmental and epileptic encephalopathies^48,85^.

The second ID candidate gene, *ETFBKMT* (electron transfer flavoprotein subunit beta lysine methyltransferase, MIM 615256) known as *METTL20* (Methyltransferase like 20), is a lysine methyltransferase. Some lysine methyltransferases such as *KMT2C* (Lysine Methyltransferase 2C, MIM 606833, aka MLL3), *SETD1B* (SET Domain Containing 1B, MIM 611055, aka KMT2G, Lysine-specific Methyltransferase 2G)^23,86^, *EHMT1* (Euchromatin Histone Lysine Methyltransferase 1, MIM 607001)^87,88^, and KMT5B (Lysine Methyltransferase 5B, MIM 610881)^89^ are well known to be associated with neurodevelopmental disorders. On the protein level, ETFBKMT interacts with TUBB2A (Tubulin, Beta-2A, MIM 615101)^90^, TUBB4A (Tubulin, Beta-4A, MIM 602662 )^90^, DARS2 (Aspartyl-tRNA Synthetase 2, MIM 610956)^55^, and GLS (Glutaminase, MIM 138280)^91^, which are associated with NDDs. *TUBB2A* is associated with seizures, ID and development delay^92^, while *TUBB4A* mutations cause leukoencephalopathy hypomyelination with atrophy of the basal ganglia and cerebellum^93^. *DARS2* is genetically linked to leukoencephalopathy with brain stem and spinal cord involvement^94,95^. Trinucleotide expansion in *GLS* causes development delay, ataxia, and cerebellar atrophy^96^.

*DDX11* (DEAD/H-Box Helicase 11, MIM 601150) associated with autosomal recessive Warsaw Breakage syndrome with intellectual disability^97^ was excluded due to its bi-allelic inheritance pattern.

Interestingly, four genes, *INTS13, PPFIBP1, REP15*, and *FAR2*, are likely candidates for KS coupled with ID at 12p11.2 (Fig. 2A).

*INTS13* (Integrator Complex Subunit 13, MIM 615079) also known as *ASUN* (Asunder, Spermatogenesis Regulator) is mapped 65 kb distal from the 12p11.23 telomeric breakpoint of the 4.7 microdeletion. Although it is not directly encompassed in the 4.7 Mb deletion at 12p11.21–12p11.23, it might be dysregulated by a positional effect^30^ in the KS phenotype seen in this patient with an unbalanced chromosome translocation^2^. Three variants in this gene are reported in NDD patients (Table 2). *INTS13* is also a critical regulator of spermatogenesis in *Drosophila melanogaster*. The study showed that knockout of this gene in Drosophila caused a defect in spermatogenesis, showing spermatocyte arrest during prophase of meiosis I^98^. Another study revealed that germline expression of mouse Asun rescued sterility and dynein mislocalization in Asun mutant flies^99^

PPFIBP1 (PPFIA Binding Protein 1, MIM 603141) has been found to interact with TACR3 (Tachykinin Receptor 3, MIM 162332)^55^, a gene mutated in patients with hypogonadotropic hypogonadism^100^. Among the interacting proteins of PPFIBP1, YWHAG (Tyrosine 3-Monooxygenase/Tryptophan 5-Monooxygenase Activation Protein, Gamma Isoform, MIM 605356)^55^, KRAS (KRAS Protooncogene, GTPase, MIM 190070)^60^, NRAS (NRAS Protooncogene, GTPase, MIM 164790) and HRAS (HRAS Protooncogene, GTPase, MIM 190020)^101^, CUL3^102^, and SNAP29 (Synaptosomal-Associated Protein, 29-KD, MIM 604202)^55^ suggest a neurodevelopmental role of *PPFIBP1*. Five missense variants in *YWHAG* were reported in patients with developmental and epileptic encephalopathy^103^, and *KRAS*^104^ and *NRAS*^105^ are associated with Noonan syndrome, whereas *HRAS* is involved in Costello syndrome^106^. Apart from distinct facial dysmorphism, both syndromes share a neurodevelopmental phenotype. *CUL3* mutations cause neurodevelopmental^77^ and autism spectrum disorder^50^. *SNAP29* is genetically associated with Cednik syndrome including neuropathy^107^, and schizophrenia^108^.

The second candidate gene *REP15* (RAB15 Effector Protein, MIM 610848) interacts with SLC4A2 (Solute Carrier Family 4, Member 2, MIM 109280)^55^. Histopathologic analysis of *Slc4a2* KO mice revealed an interruption in spermiogenesis leading to infertility^109^. Moreover, REP15 interacts effectively with TLK2 (Tousled-Like Kinase 2, MIM 608439)^55^, which is associated with neurodevelopmental delay^110^, autism spectrum disorder^58^ and schizophrenia^111^.

*FAR2* (Fatty Acyl-CoA Reductase 2, MIM 616156), the third candidate gene for KS coupled with ID, physically interacts with the zona pellucida glycoprotein 2 (*ZP2*, MIM 182888)^55^, variants of which were found in females with infertility^112–116^. FAR2 interacts with ATP2B2 (ATPase, Ca (2+)-Transporting, Plasma membrane, 2, MIM 108733)^55,59^, variants of which are found in patients with autism spectrum disorder^58,117^. KCNA2 (Potassium Channel, Voltage-Gated, Shaker-Related Subfamily, Member 2, MIM 176262) interacting with FAR2^55^, is associated with epileptic encephalopathy^118^ and epilepsy^119–122^. Another FAR2 interacting protein, CUL3, is associated with neurodevelopmental disorders^77^ and autism spectrum disorder^50^.

Collectively, *INTS13, PPFIBP1, REP15*, and *FAR2* are good candidate genes for KS combined with NDDs at 12p11.22–12p11.23.

*PTHLH* (parathyroid hormone like hormone MIM 168470) is associated with brachydactyly^123^ and explains this phenotype in the subject DCP308811. Bi-allelic variants of *IPO8* (importin 8 MIM 605600) are linked to cardiovascular defects, skeletal anomalies, and immune dysregulation^124^. We excluded both genes due to the phenotype unrelated to KS and ID.

We propose that, despite not being encompassed by small CNVs we used in comparative genomic mapping, the expression levels of the two ID candidate genes *DENND5B*, and *ETFBKMT* could be altered due to position effect^125,126^, providing a likely explanation for NDD phenotypes such as dystonia, global developmental delay, growth delay, motor delay, etc., observed in one DECIPHER proband DCP288321 (Fig. 2A and Table 3).

We also confirmed the high expression of our candidate genes in five different human tissues (*i.e*., brain, fetal brain, muscle, ovary, and testis) relevant to the phenotype of KS and NDD, to substantiate their pathogenicity (Fig. 4B).

During the composition of this manuscript, genome sequencing was performed in this same patient to map the translocation breakpoint, with the conclusion that deletion of *RMST* was implicated as a cause of KS through loss of function by the erroneous assumption that this chromosome translocation is balanced^14^. RMST physically interacts with SOX2^127^, a transcription factor known to regulate neural fate, and aids in the binding of SOX2 to the promoter of target genes important in neurogenesis^127^. *SOX2* (SRY-box transcription factor 2, MIM 184429) is a known disease gene for hypogonadotropic hypogonadism and combined pituitary hormone deficiency^1^. *RMST* has also been associated with rhabdomyosarcoma and melanoma^128^.

This ostensible pathogenicity of *RMST* in KS remains to be seen, because this subject has an unbalanced translocation accompanied by an additional 4.7 Mb microdeletion that we identified. Moreover, we did not find any mutations of this gene in the 48 KS patients we recruited. Our case underscores the necessity and significance of aCGH or sequencing analysis in individuals with disease-associated apparently balanced translocations to rule out cryptic microdeletions. At the same time, this study highlights the benefit of the integrated usage of karyotype analysis, aCGH and sequencing for an informed approach to phenotypic assessment.

In summary, we found that an apparently balanced translocation t(7;12)(q22;q24)^2,13,14^ is actually unbalanced and the 4. 7 Mb cryptic deletion at 12p11.21–12p11.23 we identified is likely to explain the phenotype of KS and ID in the subject carrying these two unrelated chromosomal rearrangements. *In silico* comparative genomic mapping with additional 14 CNVs in this genomic region identified one potential KS candidate gene (*TSPAN11*), seven candidate genes for neurodevelopmental disorder (*TM7SF3, STK38L, ARNTL2, ERGIC2, TMTC1, DENND5B, and ETFBKMT*) and four candidate genes for KS with ID (*INTS13, REP15, PPFIBP1*, and *FAR2*). The candidacy of these genes was further supported by the high-level expression pattern in the relevant human tissues. We propose that some dosage-sensitive genes, either increased or decreased, in this genomic region might contribute to the sexual and/or cognitive impairment in the patients with KS and/or ID. Among our candidate genes, the probabilities of dosage sensitivity, such as pHaplo and pTriplo of at least three genes, are high (STK38L: pHaplo/pTriplo 0.77/0.94, DENND5B: 0.96/0.96, PPFIBP1: 0.8/0.7)^129^, suggesting that both its increase or decrease can result in a related deleterious phenotype. RT-qPCR or western blot of candidate genes encompassed in both a deletion and duplication will help generate substantiating evidence for this hypothesis.

It is well known that heterogeneous neurodevelopmental phenotypes are caused by mutations in the same gene^130^. Given that our seven CNV subjects show diverse neurodevelopmental phenotypes including intellectual disability, autism, and epilepsy, our candidate genes at 12p11.21–12p11.23 will offer an opportunity to identify NDD disease genes from NGS databases containing a myriad of autosomal dominant or *de novo* VUSs.

## Materials And Methods

### Human Subjects

This study was approved by the Augusta University Institutional Review Board (IRB). Methods were carried out in accordance with the principles stated in the American Society of Human Genetics Code of Ethics. The participants were recruited by endocrinologists, gynecologists, or clinical geneticists for clinical characterization and genetic studies with unaffected siblings and parents when available. From all participants an informed consent approved by the IRB of Augusta University was obtained for the genetic studies. DNA from a cohort of 48 American probands with KS or IHH and other accompanying minor phenotypes (24 women, 24 men) were used for mutation screening of positional candidate genes identified at both genomic breakpoints. We isolated DNA from the peripheral blood of each person and 48 individuals were negative for *ANOS1* and *FGFR1* variants. The KS diagnosis was based on the presence of IHH, defined as delayed or no pubertal maturation in combination with low serum gonadotropins and low sex steroids, in combination with a smell deficit identified from the patient’s history and/or formal smell testing.

### Clinical reports

#### Subject 1– 4.7 Mb del(12)(p11.21p11.23), t(7;12)(q21.13;q23.1)

A detailed description of this DGAP032 subject with a balanced *de novo* reciprocal translocation, 46,XY,t(7;12)(q22;q24)dn was previously published in 1990^2^. In brief, the patient was a 44-year-old Chippewa/French man in 1990 with hypogonadotropic hypogonadism, based upon low levels of FSH, LH, and testosterone, along with sparse pubic hair, small testes (< 1 cm), deficiency of olfaction, skeletal and cranial anomalies, and ID. He was referred to a hospital at the age of 22 years due to delayed sexual development. During this evaluation, he was noted to have normal 17-hydroxycorticosteroids and abnormally low 17-ketosteroids and gonadotropin levels. The epiphyseal centers of most long bones and the spine were not yet closed. The bone age of the hand was in the “neighborhood” of 12 years, and the metacarpals appeared shortened and clubbed at their distal ends, especially the 4th right metacarpal, indicating brachydactyly. In addition, a sharply outlined foramen in the occipital bone near the internal occipital protuberance was noted. In 1984, lymphocyte chromosome studies demonstrated an apparently reciprocal translocation, t(7;12)(q22;q24), which was revised as t(7;12)(q21.13;q23.1)dn based on our molecular analysis result (Fig. 1A)^13^. Clinical signs of KS were not seen in his five full sisters, as well as one full brother, two half-brothers, or one half-sister.

#### Subject 2– 500 kb dup(12)(p11.23)

**Figure 5:**
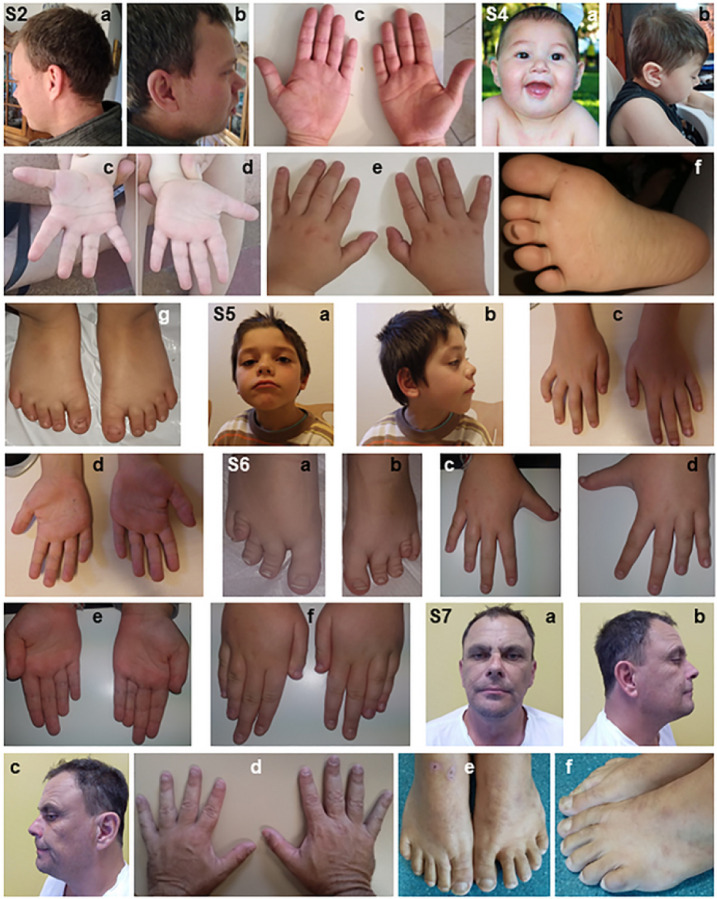
Facial and limb pictures of individuals with CNVs at 12p11.21–12p11.23. S2: Subject 2 on Table 1 (a, b) borderline rotated left and right ears (c) palms showing special form and disrupted vertical creases. S4: Subject 4 on Table 1 shows (a) microcephaly at one year (b) lateral view of right face at 3 years 10 months (c) hypoplasia of the vertical or Thenar palmar flexion creases (PFC) on the right palm (d) the vertical PFC looks short and the transverse proximal looks with some tendency to Sidney line on the left palm (e) tapering fingers (f) a minor syndactyly between toes 2 and 3 (g) small toes and a questionable gap between toes 1 and 2 and S5: Subject 5 on Table 1 shows (a) small forehead, right eyelid ptosis, bilateral downward palpebral fissures, frontal hair spike, lower lip eversion (c) tapering fingers and bilateral short 5th fingers with clinodactyly (d) the transverse proximal PFC has tendency to join the transverse distal, a variant of the PFC. S6: Subject 6 on Table 1 shows (a,b) short 4th toes likely due to short 4th metacarpals on both feet (c,d) dorsal view of tapering fingers with short 4th and 5th fingers in both hands (e) the transverse distal palmar flexion crease is rather short and doesn’t start at the ulnar margin on both palms (f) dorsal view of both hands showing bilateral short 4th fingers and short 5th fingers especially at right S7: Subject 7 on Table 1 shows (a) essentially no dysmorphism (b,c) microcephaly (d) tapering fingers (e,f) clinodactyly of the 5th toes and a gap between toes 1 and 2 in the left foot.

Subject 2 (50943) is a 36-year-old man with a history of ID, developmental delay, autism, and dyslexia. The delivery took place with a birth weight of 3500 g. His IQ, assessed at 48 months, was between 30–50. He sat independently at around 11 months, walked at 27 months, was toilet-trained at 2 years, and spoke his first words at 30 months. He was also noted to have learning disability, language delay, and speech delay. At 29 years old, he was noted to speak often loudly, and demanding frequently with incomprehensible associations. His tantrums are verbal but seldom physical and sometimes very incriminating. He is extremely restless with no tics or stuttering, constantly repeats words and parrots “yes.” He speaks very loudly. Although no apparent physical abnormalities were observed, he was noted to have hypertelorism and a slightly thicker lower lip. He did not have seizures, bone anomalies, facial dysmorphism, or shortened fingers or toes (Fig. 5). However, a brain MRI displayed an arachnoid cyst. aCGH analysis carried out on genomic DNA revealed a *de novo* 500 kb duplication, 46,XY,arr[hg 38] (chr12:27,134,884 – 27,634,952)×3dn at 12p11.23.

#### Subject 3– 750 kb dup(12)(p11.22p11.23)

Subject 3 (31606) is a 10-year-old female with a history of developmental delay, speech delay, expressive language delays, and *ADHD*. At the age of 15 months, she managed to walk; however, she had occupational therapy because she would not crawl, and she also required social/emotional therapy. She was enrolled in early childhood education and speech therapy and attended a mainstream school at 4.5 years old. She had an aversion to textured foods as a baby requiring feeding therapy. At 3 days of age, she had chronic diarrhea and did not grow or gain weight well. She was hospitalized four times from 52 days until 5 months for failure to thrive and chronic diarrhea. She was hospitalized a third time for 18 days with no conclusive diagnosis despite testing. She was chronically dehydrated because her body could not absorb necessary nutrients as fluids passed through her quickly. A second gastroenterology opinion was sought; however, no additional tests were conducted. She also had congenital sucrose-isomaltase deficiency (CSID). aCGH analysis revealed a 750 kb duplication, 46,XX,arr[hg 38] (chr12:27,157,806 – 27,907,534)×3 in 12p11.22-p11.23.

#### Subject 4– 1.94 Mb dup(12)(p11.22)

Subject 4 (022821) is a four-year-old white male with a history of learning disability, dyslexia, hypotonia, language delays, and delayed speech. Born at full-term, via normal spontaneous delivery, he crawled at 7 months, sat alone at 9 months, and walked unassisted at 2 years of age. He required time to be able to sit alone because he had no strength in his abdomen. He took his first steps at 26 months, and since 30 months, has been receiving occupational therapy (OT). Evaluated by a neurologist at 15 months, he was diagnosed as having microcephaly (OFC 43 cm, approximately – 3SD for age), short stature, developmental delay, and impaired motor skills. He also presents with syndactyly and tapering fingers. At age 2 years, some visual asymmetry in visual evoked potentials was detected. CMV and other intrauterine infections in the mother were excluded. He was always attentive to his surroundings, but with no attempt to communicate. At 3 years and 10 months, it was discovered that he had moderate hearing loss in the left ear and mild hearing loss in the right ear (Fig. 5). An EEG done at age 23 months was normal. Chromosomal analysis was normal, but a SurePrint-Ga Human Genome Kit Agilent aCGH (4×180K) revealed a 1.94 Mb duplication, 46,XY,arr[hg 38] (chr12:28,047,313 – 29,990,575)×3 in 12p11.22. The subject presents characteristics of autism spectrum, such as walking on tiptoe, repetitive movements, and obsession with spinning objects.

#### Subject 5– 215 kb dup(12)(p11.23)

Subject 5 (DCP295472) is 12 years and 5-month-old Caucasian male with a history of autism spectrum disorder and learning disability. He was born at full-term by spontaneous vaginal delivery and weighed 3.810 kg (76th centile), length of 50 cm (38th centile), and had a head circumference of 34 cm (22nd centile). He suffered from repetitive ear infections in childhood and underwent a surgery for ear tubes. He walked at 24 months and had a language delay associated with a global developmental delay and impaired motor skills. Neurobehavioral concerns began at 2 years when he displayed repetitive movements and stereotypy. He suffered from difficulties regulating emotions and had few facial expressions. Sleeping disorders occurred with early awakening at 3–4 am treated by melatonin. He expressed food selectivity. At 10 years of age, he weighed 30.8 kg (median) with a height of 135 cm (median) and head circumference of 53 cm (median). He had right eyelid ptosis. While an ophthalmic surgery was planned, the subject never came to the anesthesiologist appointment. Dysmorphic features included bilateral downward palpebral fissures, right eyelid ptosis, frontal hair spike and lower lip eversion. He also presented with clinodactyly of the 5th finger in the right hand (Fig. 5). He followed a normal schooling with Abbotsford Virtual School. His treatment was methylphenidate and melatonin. The tests completed for this patient included a chromosome Fragile X analysis and aCGH. Chromosomal analysis showed a paternally inherited 215 kb duplication, 46,XY,arr[hg 38] (chr12:27,400,730 – 27,615,518)×3 *pat* in 12p11.23 and 407 kb deletion at 2q13, arr[hg 38] (chr2: 108,684,076–109,090,916)×1 pat. The father presented with anxiety disorder like his affected son; however, follow-up with the father was limited.

#### Subject 6–2.18 Mb del(12)(p11.21p11.22)

Subject 6 (DCP370033) is an 11-year-old Caucasian girl with a history of developmental delay, speech delay, learning difficulties, and ADHD. She is the first child of a healthy and non-consanguineous couple. The pregnancy was uncomplicated. She was born full term by spontaneous vaginal delivery and weighed 3 kg (99th centile). She has two younger sisters, one of whom has a unilateral third finger brachydactyly, according to her mother. The patient sat independently at approximately 7 months, walked at 22 months, and spoke her first words late. At age 5 years, speech delay with articulation problems became apparent. Psychological tests revealed a clear discrepancy between performance and verbal capacities. The mother described behavioral challenges including temper tantrums and a short attention span. She is presently in the 3rd grade, has mild ID and learning difficulties, shows a poor attention span, and is easily distracted. She is in a special education program, with curricular adaptations. She is seen in a neuropediatric clinic for recurrent headaches that improved after withdrawing methylphenidate treatment and is in an endocrinology clinic for obesity. She has normal stature, normal head circumference and no dysmorphic features. She presents with a large thumb, shortening of the IV and V metacarpals and of the IV and V metatarsals, tapering fingers without clinodactyly or syndactyly. She has short toes and a short 4th metatarsal bone (Fig. 5). Her father with the same microdeletion had some learning difficulties and with the same abnormal feet, but he finished the 12th grade as did her uncles, who were not tested for aCGH. The father has at least two brothers with more severe learning difficulties, but they are able to live independently. aCGH (CGX-HD 180K (PerkinElmer^®^)) performed shows a paternally inherited 2.18 Mb deletion-arr[hg38] 12p11.21p11.22(chr12:28,414,984 – 30,598,365)×1 pat and 49 kb duplication with unknown inheritance at Xp22.33, arr[hg38] (chrX:1,259,698–1,308,697)×3.

#### Subject 7– 652 kb dup(12)(p11.22)

Subject 7 (DCP293962) is a 48-year-old Caucasian male with a history of developmental delay, dyslexia and ID with poor academic performance. He was born full term by spontaneous vaginal delivery with an average birth weight. His neonatal period was unremarkable. He had normal gross and fine motor milestones. He was an average child regarding his social interactions. He was diagnosed with dyslexia in early childhood and has speech delay. He could not read and can barely write his name. He left school in grade 8 and has been on a disability pension since then because he had difficulties holding a job. He does not have any disruptive or aggressive behavior. He has recently married at the age of 42. At this age, his height was 179 cm, weight was 95 kg and his head circumference was 59.2 cm. He was dysmorphic showing upturned nostrils and a high nasal bridge. He presents clinodactyly and tapering fingers (Fig. 5). The subject’s examination was unremarkable. His fragile X and urine metabolic screen were normal. His aCGH showed a paternally derived microduplication: arr[hg38] 12p11.22 dup (chr12:28,701,107 – 29,353,047)×3 *pat* and 229 kb deletion with unknown inheritance at 16p13.3, arr[hg38] (chr16:6,699,348–6,927,950)×1. The father had mild learning problems with the same syndactyly and tapering fingers.

### Eight Decipher CNV patients in Fig. 2A

Brief clinical information, genomic coordinates, and inheritance patterns of eight Decipher CNV patients used *in silico* comparative genomic mapping in Fig. 2A are described in Table 3.

#### Fluorescence in situ hybridization analysis

A lymphoblastoid cell line (GM10565) from Subject 1, designated DGAP032 in the Developmental Genome Anatomy Project, was obtained from the NIGMS Human Genetic Cell Repository at the Coriell Institute for Medical Research (www.coriell.org)^12^. The karyotype, 46,XY,t(7;12)(q22;q24)dn, was reconfirmed prior to refinement of the breakpoint by FISH. Assignment of chromosome breakpoint locations to chromosomal bands was determined by GTG-banding. To identify genes potentially disrupted in the subject, translocation breakpoints were mapped using FISH. Maps from the National Center for BiotechnoIogy Information (http://www.ncbi.nlm.nih.gov/genome/guide/human/)^131^ and the Genome Bioinformatics Group at the University of California Santa Cruz (http://genome.ucsc.edu/)^132^. Guided selection of BAC clones for breakpoint mapping was done by FISH. BAC clones from the RP11 (Children’s Hospital of Oakland Research Institute) and the CIT pool D (Research Genetics) libraries corresponding to relative locations on the UCSC map from chromosomes 7 and 12 were used as FISH probes on metaphase chromosome spreads from an Epstein-Barr virus-transformed lymphoblast cell line generated from the subject`s peripheral blood. Metaphase spreads were prepared according to a standard cytogenetic protocol. Human BAC clones were obtained from the RP11 (Children’s Hospital of Oakland Research Institute) and the CIT pool D (Research Genetics) libraries. BAC DNA was purified by alkaline lysis and isopropanol precipitation. After purification, BAC DNA was directly labeled by nick-translation with either SpectrumOrange or SpectrumGreen labeled nucleotides (Vysis) and used in single- or two-color FISH experiments. Slides were counterstained with 4’,6’-diamidino-2-phenylindole hydrochloride (DAPI). Representative metaphase images were recorded using the CytoVision image analysis system (Applied Imaging) database.

Using the relative STS positions on the UCSC map, BAC clones were chosen to cross the relevant regions on chromosomes 7 and 12. FISH analysis of each clone was then used to identify clones that mapped proximal or distal to each chromosome breakpoint. By this way, physical maps of chromosomes 7q21 and 12q24 were constructed, and the breakpoint regions narrowed and defined^13^.

### Southern blot analysis

Southern blot analysis of patient lymphoblast genomic DNA with seven probes (from KS-1 to KS-7) to search for altered restriction fragments was carried out using standard protocols (Fig. 3B). For each lane, 10 μg of genomic DNA from the patient and control were digested with an appropriate restriction enzyme. Fragments were separated on a 1.0% agarose gel and transferred to Hybond-N membrane (Amersham, Arlington Heights, Illinois, USA). Filters were ultraviolet cross linked, baked at 80°C, and hybridized with probes labelled with 32P-dCTP by random priming. Hybridization of labelled fragments was done in the presence of excess herring sperm competitor DNA, and hybridized membranes were washed at 60°C with 0.15 M NaCl/0.015 M sodium citrate/0.1% sodium dodecyl sulphate (SDS) for 30 minutes. Autoradiography took place for 16 hours at − 70°C using two intensifying screens. Seven hybridization probes were amplified by the primer sets mentioned in Supplementary table 1. After the breakpoint region was apparently narrowed to 3.5 kb between CTD-2268E11 and CTD-2542D2 at chromosome 12 in band q23 by FISH, the first four genomic probes, KS-1, KS-2, KS-3, and KS-4, within this region were amplified from the breakpoint spanning BAC clone RP11–492N15 under the following conditions (Fig. 1B, Fig. 2B, and Fig. 3A)∷ initial denaturation at 94°C for 2 min, followed by 30 cycles at 94°C for 30 sec, 58°C for 30 sec, 72°C for 45 sec (KS-1, KS-2, and KS-3) or 3 min for 30 sec (KS-4), and extension at 72°C for 5 min after the last cycle. Two genomic probes KS-5 and KS-6 were amplified from this region (Fig. 3A) using BAC RP11–492N15 under the following conditions: initial denaturation at 94°C for 2 min, followed by 30 cycles at 94°C for 30 sec, 63°C for 30 sec, 72°C for 50 sec, and extension at 72°C for 5 min after the last cycle. The probe KS-7 was amplified within that putative breakpoint region using BAC RP11–492N15 under the following conditions: initial denaturation at 94°C for 2 min, followed by 30 cycles at 94°C for 30 sec, 58°C 30 sec, 72°C for 40 sec, and extension at 72°C for 5 min after the last cycle. The list of primers used for the ampli cation of probes for Southern hybridization is presented in Supplementary Table 1.

### Suppression PCR and Nested PCR

The 3.5 kb junction fragment from der(12) was amplified by suppression PCR using the following primer sets and the conditions. Primers flanking the 1.5 kb narrowed breakpoint region from the 12q23.1 were used with adaptor-based primers: PCR1): AP1-A 5’CCTAATACGACTCACTATAGG3’ + AC007351–54209rev 5’GTGAATGGTGGATAGTGCTC3’; AP2-A 5’CTATAGGGCTCGAGCGGC3’ + AC007351–54176rev 5’GATTAAATTCACTCTCTGAAGAA3’. PCR2): AP1-A 5’CCTAATACGACTCACTATAGG3’ + AC007351–54176rev 5’ GATTAAATTCACTCTCTGAAGAA3’; AP2-A 5’CTATAGGGCTCGAGCGGC3’ + AC007351–54026rev 5’CTAGCTTACAATTTTCTGGTGA3’. Initial denaturation was at 94°C for 2 min, followed by 30 cycles at 94°C for 30 sec, 57°C for 30 sec, 72°C for 1 min 30 sec, and extension at 72°C for 5 min after the last cycle.

The 2.3 kb junction fragment from der(7) was amplified by nested PCR using the following primer sets and the conditions. Initial denaturation at 94°C for 2 min, followed by 30 cycles at 94°C for 30 sec, 57°C for 30 sec, 72°C for 2 min 30 sec, and extension at 72°C for 5 min after the last cycle. PCR1)^13^: 5’CCATTGGCTTTAAGTGTATAGT3’+ 5’CTTGTGTGTACATCTCCTGAA3’; PCR2): 5’CAACAGACATCTGCATTTACTT3’+5’GAAGATAGCTATAACAACAGC3’.

### Mutation screening of five genes at the breakpoints of t(7;12) (q21.13;q23.1)

We screened for mutations in five genes - *RMST, NEDD1, PAFAH1B2P2, ZNF804B*, and *STEAP4* - in 48 KS patients including our translocation subject, who was also screened for *FGFR1* (Fibroblast Growth Factor Receptor 1, MIM 136350) and *ANOS1* (Anosmin 1, MIM 300836) to exclude the possibility of mutations in these two well-known genes with high prevalence for KS. A combination of single strand conformation polymorphism analysis (SSCP) and direct sequencing of *RMST* and *ZNF804B* were performed in mutation screening. The 46 primer sets were designed to cover all exons and flanking intronic regions of two predicted mRNAs. The size of amplicon is adjusted to less than 350 bp for SSCP, and a few parts of mRNAs were applied on PCR and direct sequencing to check for a mutation. PCR was carried out with 10 ng of genomic DNA of the subject’s sample or normal control in 20 ul of reaction (primer sequences and amplification conditions are available on request). Then PCR products were electrophoresed on precast gels of ExcelGel DNA Analysis Kit (Amersham Biosciences) according to the manufacturer’s instructions. Sequentially, the DNA gel was stained by silver stain according to the manufacturer’s instructions (DNA silver staining kit; Amersham Biosciences) to visualize and permanently stain the discrete DNA bands. When aberrant band patterns were recognized on the samples compared with normal controls, PCR products that have the aberrant band on the gel were sequenced with ABI Prism 377 sequencer (Applied Biosystems, Foster City, Calif.). Sequences were aligned and compared with sequences of predicted mRNAs to confirm the mutation. For seven genes, all coding regions and exon-intron boundaries were directly amplified and sequenced. NCBI reference mRNA sequences used for screening were NR_152618.1 (*RMST*), NM_001135175.1 (*NEDD1*), NR_077240.1 (*PAFAH1B2P2*), NM_181646.5 (*ZNF804B*), NM_024636.4 (*STEAP4*), NM_001174067.1 (*FGFR1*), and NM_000216.4 (*ANOS1*).

### aCGH (Array Comparative Genomic Hybridization)

DNA extracted from the cell line was compared to a reference sample for standard two-color aCGH. Reference DNA was purchased from Promega (Madison, WI, USA). Test samples were labeled using Cy5 and reference DNA was labeled using Cy3. Agilent 244K human genome oligonucleotide aCGH (G4411B) was used for aCGH analysis following the manufacturer’s instructions (Oligonucleotide Array-Based CGH for Genomic DNA Analysis protocol version 3 (Agilent Technologies, Palo Alto, CA, USA). Images were captured using an Agilent scanner and quantified using Feature Extraction software v9.0 (Agilent Technologies, Palo Alto, CA, USA). CGH analytics software v3.4 (Agilent Technologies, Palo Alto, CA, USA) was subsequently used for data normalization, quality evaluation, and data visualization. Copy number aberration was indicated using the ADM-2 (Aberration Detection Method 2) algorithm. Probe positions were mapped to GRCh38.

#### In Silico Comparative CNV mapping

The phenotypes from our seven CNV patients (Subjects 1–7), DGAP032, 50943, 31606, 022821, 295472, 370033 and 293962 (Table 1) were compared with eight unpublished CNV cases from the DECIPHER database. Genomic coordinates from these cases were converted to hg38 before the comparison was carried out (Table 3). Three factors were considered for choosing candidate genes – (1) sporadic genetic variants reported in humans with matching phenotypes, (2) knockdown or knockout animal models recapitulated human phenotypes, (3) their interacting proteins were investigated, and the genetic variants of corresponding genes reported in human patients with a similar phenotype (Table 2). Literature was also reviewed as well as several databases including Human Gene Mutation Database HGMD Professional (2022.2) (https://my.qiagendigitalinsights.com/bbp/view/hgmd/pro/start.php), MGI (6.21) (Mouse Genome Informatics) (http://www.informatics.jax.org/), BioGrid (4.4.212) (Database of Protein, Chemical, and Genetic Interactions, thebiogrid.org), and VarElect (https://varelect.genecards.org/).

### Quantitative Reverse Transcription PCR (RT-qPCR)

RT-qPCR was performed from total RNA of five different human tissues including brain, fetal brain, muscle, ovary and testis. Catalog numbers of the five tissues obtained from Clontech were as follows: brain- 636530, fetal brain total-636526, muscle- 636534, ovary- 636555 and testis- 636533. cDNA synthesis was performed using 1–2 μg of total RNA using high-Capacity cDNA Reverse Transcription Kit and analyzed by RT-PCR on QuantStudio 6 Flex system using SYBR Green (ThermoFisher, Waltham, MA). The ΔCt method was used to calculate the relative expression of each gene. In conclusion, the difference between the Ct values (Ct) of the target gene and the reference gene, *GAPDH*, was used to compute relative gene expression. After determining ΔCt, the fold change (2 – ΔCt) was calculated, and the relative expression was plotted as excel graphs.

## Figures and Tables

**Table 1:** Individual clinical features of seven subjects with 12p11.2 heterozygous deletions/duplications along with their demarcating genomic coordinates (hg38).

Gene MIM#	Gene name	Chrom. location	Function	Reported phenotype and remarks
*INTS13* (aka ASUN) 615079	integrator complex subunit 13	12p11.23	involved in regulation of mitotic cell cycle	*de novo* nonsense variant C.118G > T (NM_018164.3) p.G40* (NP_060634.2) in autism spectrum disorder (Kosmicki et al., 2017) *de novo* synonymous variant C.672A > G (NM_018164.3) p.K224= (NP_060634.2) in neurodevelopmental disorder (Turner et al., 2019) nonsense variant c.295C > T (NM_018164.3) p.Q99* (NP_060634.2) in intellectual disability (Gilissen et al., 2014) critical regulator of spermatogenesis in Drosophila (Anderson et al., 2009) germline expression of mouse Asun rescued sterility and dynein mislocalization in Asun mutant flies (Jodoin et al., 2012)
*TM7SF3* 605181	transmembrane 7 superfamily, member 3	12p11.23	involved in the inhibition of cytokine-induced death of pancreatic beta cells	*de novo* missense variant C.436G > C (NM_016551.3) p.D146H (NP_057635.1) in developmental disorder (Deciphering Developmental Disorders, 2017;Turner et al., 2019) TM7SF3 is interacting with HNRNPL (Fei et al., 2017), a gene involved in intellectual disability (Hu et al., 2019)
*STK38L* 615836	serine/threonine protein kinase 38-like protein	12p11.23	involved in the regulation of structural processes in differentiating and mature neuronal cells	*de novo* nonsense variant C.889C > T (NM_015000.4) p.Q297* (NP_055815.1) in autism spectrum disorders (Kosmicki et al., 2017) candidate gene for schizophrenia C.546A > C (NM_015000.4) p.K182N (NP_055815.1) (Fromer et al., 2014) arbor-specific changes in dendritic complexity seen in the hippocampus of Stk38l KO mice (Rehberg et al., 2014)
*ARNTL2* 614517	aryl hydrocarbon receptor nuclear translocator-like protein 2	12p11.23	transcriptional activator, which forms a core component of the circadian clock	missense variant in autism spectrum disorder with sleep disorders c.1418T > C (NM_020183.5) p.L473S (NP_064568.3) (Yang et al., 2016) nonsense variant c.1858G > T (NM_020183.5) p.E620* (NP_064568.3) for in developmental and epileptic encephalopathy (Takata et al., 2019) ARNTL2 is interacting with CTTNBP2 (Luck et al., 2020), UBE3A (Shi et al., 2015), and PER2 (Huttlin et al., 2021), three genes involved in neurodevelopmental disorders
*PPFIBP1* 603141	protein-tyrosine phosphatase, receptor-type, f polypeptide-interacting protein-binding protein 1	12p11.22-p11.23	may regulate the disassembly of focal adhesions	novel candidate gene for congenital microcephaly in humans c.960_961delGA (NM_001198915.2) p.Glu320Aspfs*3 (NP_001185844.1) (Shaheen et al., 2019) PPFIBP1 is interacting with TACR3 (Huttlin et al., 2021), a gene involved in hypogonadotropic hypogonadism with or without anosmia (Topaloglu et al., 2009) PPFIBP1 is interacting with YWHAG (Huttlin et al., 2021), KRAS (Go et al., 2021), NRAS (Kovalski et al., 2019), HRAS (Kovalski et al., 2019), CUL3 (Kouranti et al., 2022), and SNAP29 (Huttlin et al., 2021), six genes involved in neurodevelopmental disorders
*REP15* 610848	Rab15 effector protein	12p11.22	regulates transferrin receptor recycling from the endocytic recycling compartment	*Rep15* KO Mice have an abnormal behavior phenotype (http://www.informatics.jax.org/marker/MGI:1913782) REP15 is interacting with TLK2 (Huttlin et al., 2021), a gene involved in neurodevelopmental disorder (Reijnders et al., 2018), autism spectrum disorder (Takata et al., 2018), intellectual disability (Lelieveld et al., 2016), and Schizophrenia (Gulsuner et al., 2013) REP15 is interacting with SLC4A2 (Huttlin et al., 2021), histopathologic analysis of Slc4a2 KO Mice revealed an interruption in spermiogenesis leading to infertility (Medina et al., 2003)
*FAR2* 616156	fatty acyl coa reductase 2	12p11.22	catalyzes the reduction of saturated but not unsaturated C16 or C18 fatty acyl-CoA to fatty alcohols	*PEX19*, an interactor of *FAR2* (Huttlin et al., 2015;Huttlin et al., 2017;Huttlin et al., 2021), *de novo* missense variants C.352G > A (NM_002857.4) p.D118N (NP_002848.1) (Turner et al., 2019), and c.847C > T (NM_002857.4) p.L283F (NP_002848.1) (Deciphering Developmental Disorders, 2017;Turner et al., 2019) were identified for the phenotype of developmental disorder two nonsense variants c.2133C > A (NM_001683.5) p.C711* (NP_001674.2) (Takata et al., 2018), and C.3056G > A (NM_001683.5) p.W1019* (NP_001674.2) (Takata et al., 2018) were identified in *ATP2B2*, an interactor of *FAR2* (Huttlin et al., 2017;Huttlin et al., 2021) in patients with autism spectrum disorder FAR2 is interacting with ZP2 (Huttlin et al., 2021), which is related to oocyte maturation defect leading to female infertility (Dai et al., 2019). Homozygous Zp2 −/− mouse females were sterile (Liu et al., 2017) FAR2 is interacting with GRPR (Huttlin et al., 2017), a gene disrupted at the breakpoint in a patient 46,XX,t(X;8)(p22.13;q22.1) with autism and multiple exostoses (Ishikawa-Brush et al., 1997) FAR2 is interacting with KCNA2 (Huttlin et al., 2021), and CUL3 (Kouranti et al., 2022), two genes involved in neurodevelopmental disorders grpr-deficient KO Mice showed decreased inhibition of principal neurons by the interneurons, enhanced long-term potentiation (LTP), and greater and more persistent long-term fear memory (Shumyatsky et al., 2002)
*ERGIC2* 612236	endoplasmic reticulum-Golgi intermediate compartment protein 2	12p11.22	possible role in transport between endoplasmic reticulum and Golgi	*de novo* c.945dupT (NM_016570.3) p.Val316Cysfs*26 (NP_057654.2) in autism spectrum disorder (Takata et al., 2018) ERGIC2 is interacting with SLC39A8 (Huttlin et al., 2017;Huttlin et al., 2021), CUX1 (Go et al., 2021), RAB3GAP1 (Go et al., 2021), and RAB3GAP2 (Go et al., 2021), four genes involved in neurodevelopmental disorders
*TMTC1* 615855	transmembrane and tetratricopeptide repeat domains-containing protein 1	12p11.22	transfers mannosyl residues to the hydroxyl group of serine or threonine residues	*de novo* missense variant C.932G > A (NM_175861.3) p.G311D (NP_787057.2) in development disorder (Deciphering Developmental Disorders, 2017;Turner et al., 2019) TMTC1 is interacting with BCOR (Oliviero et al., 2015), and VIRMA (Yue et al., 2018), two genes involved in neurodevelopmental disorders
*TSPAN11*	tetraspanin 11	12p11.21	integral membrane protein, regulating cell adhesion, motility, and synapse formation, interacts with integrins	missense variant C.203G > A (NM_001080509.3) p.G68D (NP_001073978.1) found in Kallmann syndrome patient (Quaynor et al., 2016)
*DENND5B* 617279	denn domain-containing protein 5b	12p11.21	guanine nucleotide exchange factor (GEF) which may activate RAB39A and/or RAB39B	*de novo* missense variant c.941C > G (NM_144973.4) p.T314S (NP_659410.3) in autism spectrum disorder (Iossifov et al., 2014;Kosmicki et al., 2017;Turner et al., 2019) DENND5B is interacting with RAB11A (Go et al., 2021), and GRB10 (Havugimana et al., 2012), two genes involved in neurodevelopmental disorders
*ETFBKMT* 615256	electron Transfer Flavoprotein Subunit Beta Lysine Methyltransferase	12p11.21	enables heat shock protein binding activity and protein-lysine N-methyltransferase activity	*de novo* synonymous variant C.783G > A (NM_173802.4) p.Q261= (NP_776163.1) in neurodevelopmental disorder (Turner et al., 2019) ETFBKMT is interacting with TUBB2A (Cloutier et al., 2013), TUBB4A (Cloutier et al., 2013), DARS2 (Huttlin et al., 2021), and GLS (Huttlin et al., 2015), four genes involved in neurodevelopmental disorders

**Table 2:** Twelve autosomal dominant positional candidate genes identified by *in silico* CNV mapping at 12p11.21–12p11.23. They include one gene for Kallmann syndrome (KS), seven genes for neurodevelopmental disorders (NDDs), and four genes for KS coupled with NDDs. The candidacy of these genes was substantiated by their genomic positions in CNV mapping, sporadic variants reported in them and their interactors in NDDs, their physical interaction with known neurodevelopmental genes, and KO mice phenotype based on HGMD, BioGrid, and MGI. Due to the large number of interacting genes, only a limited number of them in NDDs were described. KO mice data with neurobehavioral phenotype is also mentioned wherever available.

DECIPHER ID	Copy Number Variation (size)	Cytogenetic Band	Genomic Coordinates [hg38]	Inheritance	Phenotype
DCP401302	duplication (500 kb)	12p11.23	27,134,884–27,634,952	*de novo*. Additional 318 kb paternally inherited duplication at 18q12.3 (chr18:39,603,398 – 39,921,166)X3, hg38	arrhythmia, autistic behavior, hypertelorism, intellectual disability, mitral regurgitation, thick lower lip vermilion
DCP282722	deletion (454 Kb)	12p11.23	27,153,357–27,607,134	*de novo*	cleft palate, microcephaly, secundum atrial septal defect, small weight between 0.4th and 2nd centiles
DCP331121	duplication (296 kb)	12p11.23	27,153,357–27,449,723	maternally inherited	seizures, epilepsy, prepubertal
DCP331701	duplication (429 kb)	12p11.23	27,178,339–27,607,134	maternally inherited	dystonia, mild developmental delay, no dysmorphism, prepubertal
DCP308811	duplication (70 Kb)	12p11.22	27,929,322–27,999,621	maternally inherited	radial bowing, short humerus, brachydactyly type A1
DCP367151	duplication (2.51 Mb)	12p11.21–12p11.23	29,151,249–31,658,390	unknown	autistic behavior, behavioral abnormality, intellectual disability, macrocephaly, poor fine motor coordination
DCP288575	deletion (175 Kb)	12p11.22	29,755,672–29,930,757	paternally inherited. Additional four CNVs interpreted likely benign	autistic behavior, normal intelligence, autism spectrum disorder, prepubertal
DCP288321	deletion (112 Kb)	12p11.21	31,128,897–31,240,772	unknown. Additional 1.4 Mb duplication with unknown inheritance at 17p12 (chr17:14,194,981 – 15,538,864)X3, hg38	dystonia, Charcot-Marie-Tooth type 1A, prepubertal

**Table 3.** Summary of the eight heterozygous DECIPHER CNV cases at 12p11.21–12p11.23 with cytogenetic location, inheritance pattern, and brief phenotypic description. Genomic coordinates from all cases were reviewed and converted to hg38.

DECIPHER ID	Copy Number Variation (size)	Cytogenetic Band	Genomic Coordinates [hg38]	Inheritance	*Phenotype*
DCP401302	duplication (500 kb)	12p11.23	27,134,884–27,634,952	de novo. Additional 318 kb paternally inherited duplication at 18q12.3 (chr18:39,603,398–39,921,166)X3, hg38	*arrhythmia, autistic behavior, hypertelorism, intellectual disability, mitral regurgitation, thick lower lip vermilion*
DCP282722	deletion (454 Kb)	12p11.23	27,153,357–27,607,134	***de novo***	*cleft palate, microcephaly, secundum atrial septal defect*, small weight between 0.4^th^ and 2^nd^ centiles
DCP331121	duplication (296 kb)	12p11.23	27,153,357–27,449,723	maternally inherited	*seizures*, epilepsy, prepubertal
DCP331701	duplication (429 kb)	12p11.23	27,178,339–27,607,134	maternally inherited	*dystonia*, mild developmental delay, no dysmorphism, prepubertal
DCP308811	duplication (70 Kb)	12p11.22	27,929,322–27,999,621	maternally inherited	*radial bowing, short humerus, brachydactyly type A1*
DCP367151	duplication (2.51 Mb)	12p11.21–12p11.23	29,151,249–31,658,390	unknown	*autistic behavior, behavioral abnormality, intellectual disability, macrocephaly, poor ne motor coordination*
DCP288575	deletion (175 Kb)	12p11.22	29,755,672–29,930,757	paternally inherited. Additional four CNVs interpreted likely benign	*autistic behavior*, normal intelligence, autism spectrum disorder, prepubertal
DCP288321	deletion (112 Kb)	12p11.21	31,128,897–31,240,772	unknown. Additional 1.4 Mb duplication with unknown inheritance at 17p12 (chr17:14,194,981–15,538,864)X3, hg38	dystonia, Charcot-Marie-Tooth type 1A, prepubertal

## Data Availability

The genomic coordinates and phenotypes of seven patients with CNVs are listed in Table 1. Out of those, three CNVs of Subjects 2, 3, and 4, which were not previously reported, were submitted and are available at the Leiden Open Variation Database (https://www.lovd.nl/3.0/home) under the individual ID numbers 00433002, 00433003, and 00433004 with two links below. https://databases.lovd.nl/shared/individuals?search_Individual/Reference=Kim%202023 and https://databases.lovd.nl/shared/variants?search_VariantOnGenome/Reference=Kim%202023

## Abbreviations

KS: Kallmann syndrome
CNV: copy number variation
NDDs: neurodevelopmental disorders
IHH: idiopathic hypogonadotropic hypogonadism
ID: intellectual disability
aCGH: array comparative genomic hybridization
CFA: craniofacial anomalies
GnRH: hypothalamic gonadotropin releasing hormone
BAC: bacterial artificial chromosome
FISH: fluorescence *in situ* hybridization
